# High Incidence of EGFR Mutations in Pneumonic-Type Non-Small Cell Lung Cancer

**DOI:** 10.1097/MD.0000000000000540

**Published:** 2015-02-27

**Authors:** Jun Liu, Jianfei Shen, Chenglin Yang, Ping He, Yubao Guan, Wenhua Liang, Jianxing He

**Affiliations:** From the Department of Cardiothoracic Surgery, the First Affiliated Hospital of Guangzhou Medical University (JL, JS, CY, PH, YG, WL, JH); Guangzhou Institute of Respiratory Disease and China State Key Laboratory of Respiratory Disease (JL, JS, CY, PH, YG, WL, JH); Department of Pathology (PH); and Department of Radiology, the First Affiliated Hospital of Guangzhou Medical University, Guangzhou, China (YG).

## Abstract

To retrospectively identify computed tomography (CT) features that correlate with epidermal growth factor receptor (EGFR) mutation in surgically resected pneumonic-type lung cancer (P-LC).

A total of 953 consecutive patients with surgically resected lung cancer in the First Affiliated Hospital of Guangzhou Medical University from August 2011 to August 2013 were studied. The CT manifestations were reevaluated independently by 2 radiologists. The presence of pneumonic-type consolidation with pathological confirmed non-small lung cancer (NSCLC) was defined as P-LC. EGFR mutation was determined by direct DNA sequencing or amplification refractory mutation system-PCR. EGFR mutation rates as well as clinical and pathological manifestations between P-LC and control lung cancer patients were compared.

P-LC was diagnosed in 85 patients. Among these patients, 82 were adenocarcinoma (including 78 cases of invasive adenocarcinoma and 4 cases of microinvasive adenocarcinoma), 2 were squamous carcinoma and 1 was other type. P-LC occurred more frequently in female (58.8% vs 37.1%, *P* < 0.01), nonsmoking (76.5% vs 56.5%, *P* = 0.001) and adenocarcinoma (58.8% vs 37.1%, *P* < 0.01) patients. Moreover, EGFR mutations were found in 39 of 52 P-LC patients (75%) and 263 of 542 non-P-LC NSCLC patients (48.5%). However, no difference was found on the mutation sites of EGFR. Histological type, sex, and radiological manifestations (P-LC vs non-P-LC) but not smoking or sequencing method can be served as the independent predictor of EGFR mutations.

P-LC patients showed a significant higher incidence of EGFR mutations, which was independent of sex, histological type, and smoking history. The patients with imaging manifestation of pneumonic-type consolidation are highly suggested to perform EGFR mutation analysis to guide the sequential treatment.

## INTRODUCTION

Lung cancer is the most common fatal malignancy worldwide and approximately 80–90% of cases involve non-small cell lung cancer (NSCLC).^1^ NSCLC is divided into several histological subtypes, including squamous cell carcinoma, adenocarcinoma, large cell undifferentiated carcinoma, etc. Smoking is not only associated with the incidence and mortality of lung cancer, but also with the frequency of the different histological types. For example, recently we have seen a decrease in the frequency of squamous cell carcinoma and an increase in the frequency of adenocarcinoma specifically in nonsmoking individuals.^2^

Pneumonic-type lung cancer (P-LC), also known as consolidation-type lung cancer, is defined as nonobstructive diffuse solid infiltrated lung carcinoma on histology and imaging, and is usually misdiagnosed as inflammatory pulmonary consolidation. The pathological manifestation of this specific type of lung cancer, shown by only few studies, was characterized by frequent female occurrence, low correlation with smoking, lepidic growth, and dispersed alveolar consolidation.^3,4^ Even still, little is known about the cause and clinical manifestation of P-LC.

Epidermal growth factor receptor (EGFR) is a receptor with tyrosine kinase, and activating mutations in its tyrosine kinase domain promote several oncogene-driven malignancies in NSCLC. Studies have shown that (the) combination (of) chemotherapy with EGFR tyrosine kinase inhibitors could be given to patients as first-line treatment.^5,6^ It is, therefore, important to assess the EGFR mutation in the patients with NSCLC. Characteristics of EGFR mutations include frequent female occurrence, nonsmoker inclination, and a higher frequency of mutation in Asians than in Westerners.^7^ A recent epidemiology study of EGFR mutations in Asian patients with advanced NSCLC showed that the mutation frequency was 43% in Asian patients, 52% of those being Chinese patients.^8^ Most recently, Nakamura et al^9^ reported that positive EGFR mutation status may be associated with longer volume doubling time in NSCLC patients. In our study, we found that P-LC patients have significant higher incidence of EGFR mutations, independent of sex, histological type, and smoking history.

## PATIENTS AND METHODS

### Patients

The clinical data and imaging manifestations obtained from 1214 consecutive patients who underwent lung cancer resection surgery at the First Affiliated Hospital of Guangzhou Medical University from August 2011 to August 2013 were retrospectively reviewed. All the patients received resection surgery lymphadenectomy if lung cancer was confirmed by intraoperative frozen pathology; mediastinal lymphadenopathy was carried out as described by Osarogiagbon et al.^10^ Histology type of each patient was classified according to the current standard of pathological diagnosis of lung cancer issued by World Health Organization (WHO). Two hundred nine non-NSCLC and 52 cases of patient with wedge resection and open lung biopsy were excluded. A total of 953 cases were included in the study. The study protocol was approved by the Ethics Committee of the First Affiliated Hospital of Guangzhou Medical University.

### CT Findings

**FIGURE 1:**
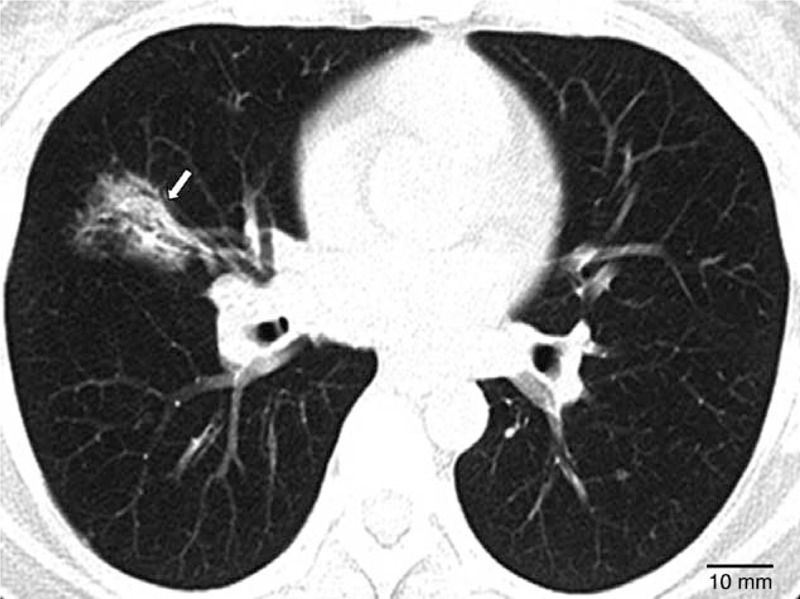
CT images showing pneumonia-like consolidation (homogenous opacity) characterized by little or no loss of volume, effacement of blood-vessel shadows, and presence of an air bronchogram. CT = computed tomography.

All patients received a chest computed tomography (CT). Two radiologists independently interpreted CT images of P-LC without knowledge of EGFR status; any discrepancies were resolved by discussion until consensus was reached. Eighty-five patients with NSCLC were included according to following criteria: histologically or cytologically proven pulmonary carcinoma, in the absence of a prior diagnosis of thoracic or extrathoracic carcinoma; CT results showed a pneumonia-like consolidation, defined as an essentially homogenous opacity in the lung defined by little or no less volume, effacement of blood vessel shadows, and, sometimes, by the presence of an air bronchogram (Figure 1); and no concomitant bacterial pneumonia or obstructive pneumonia due to an exophytic lesion occluding the lumen of the main or lobar bronchi. Exclusion criteria were presence of lobe or lung atelectasis and presence of a prior diagnosis of thoracic adenocarcinoma in 5 years.

### Histology Diagnosis

Pathologic specimens were interpreted by 2 independent pathologists according to the criteria of any differences in results were resolved by discussion until consensus was reached. Lung adenocarcinoma was determined according to the classification criteria of lung adenocarcinoma issued in 2011.^11^ Adenocarcinoma in situ (AIS) is a localized small (≤3 cm) adenocarcinoma with growth restricted to neoplastic cells along preexisting alveolar structures (lepidic growth), lacking stromal, vascular, or pleural invasion. Minimally invasive adenocarcinoma (MIA) is a small, solitary adenocarcinoma (≤3 cm) with a predominantly lepidic pattern and ≤5 mm invasion in greatest dimension in any one focus. Invasive adenocarcinoma showed lepidic, acinar, papillary, solid growth pattern and variant invasive adenocarcinoma.

### EGFR Mutation Analysis

Resected tumor specimens were used for EGFR mutation analysis. All specimens were formalin fixed and paraffin embedded. Genomic DNA were extracted from the tumor tissue and EGFR mutations in exons 18 (G719), 19 (deletion), 20 (T790), and 21 (L858 and L861)^12,13^ were detected by direct sequencing with polymerase chain reaction products (for the sample collected from August 2011 to February 2012) or amplification refractory mutation system-PCR (for the sample collected from March 2012 to August 2013). All EGFR mutation analysis was performed in the clinical laboratory.

### Collection of the Patient Data

Baseline characteristics of all patients included in the study were collected, including age, sex, smoking history, and medical history. The final pathologic lung cancer stages were assessed according to the seventh edition Union for International Cancer Control and American Joint Committee on Cancer TNM classification.^14^ The EGFR status and type of EGFR mutation, if present, were also collected.

### Statistical Analysis

All data were analyzed using SPSS software 16.0 (SPSS Inc, Chicago, IL). Enumeration data were analyzed with χ^2^ test or Fisher's exact test, as appropriate. Logistic regression model was performed on variables representing important clinical factors. *P* < 0.05 was considered as statistically significant.

## RESULTS

### Patients

Between August 2011 and August 2013, 1214 patients who underwent surgical resection lung cancer were retrospectively screened; a total of 953 cases were included in the study after excluding 261 patients with small cell lung cancer, neuroendocrine lung tumor, open lung biopsy, or wedge resection. Finally, 85 (8.9%) patients that conformed to the diagnosis P-LC criteria were found. A control group of 868 patients who underwent a homochronous operation were set as control group, including 666 (69.9%) cases of lung adenocarcinoma, 185 (19.4%) cases of squamous cancer, and 102 (10.7) cases of other type of lung cancer. EGFR mutations analysis was performed in 542 cases.

The epidemiologic data of patients from 2 groups were summarized in Table 1. The mean age of patients from these 2 groups was 59.9 years. Our study showed that more frequent occurrence of P-LC was found in patients that were female (58.8% vs 37.1%; *P* < 0.01), nonsmokers (76.5% vs 56.5%; *P* = 0.001), and diagnosed lung adenocarcinoma (96.5% vs 67.3%; *P* < 0.01). However, there was no significant difference between the P-LC and control groups in regards to the preoperative carcinoembryonic antigen levels (19.8% vs 20.2%; *P* = 0.055).

**TABLE 1:**
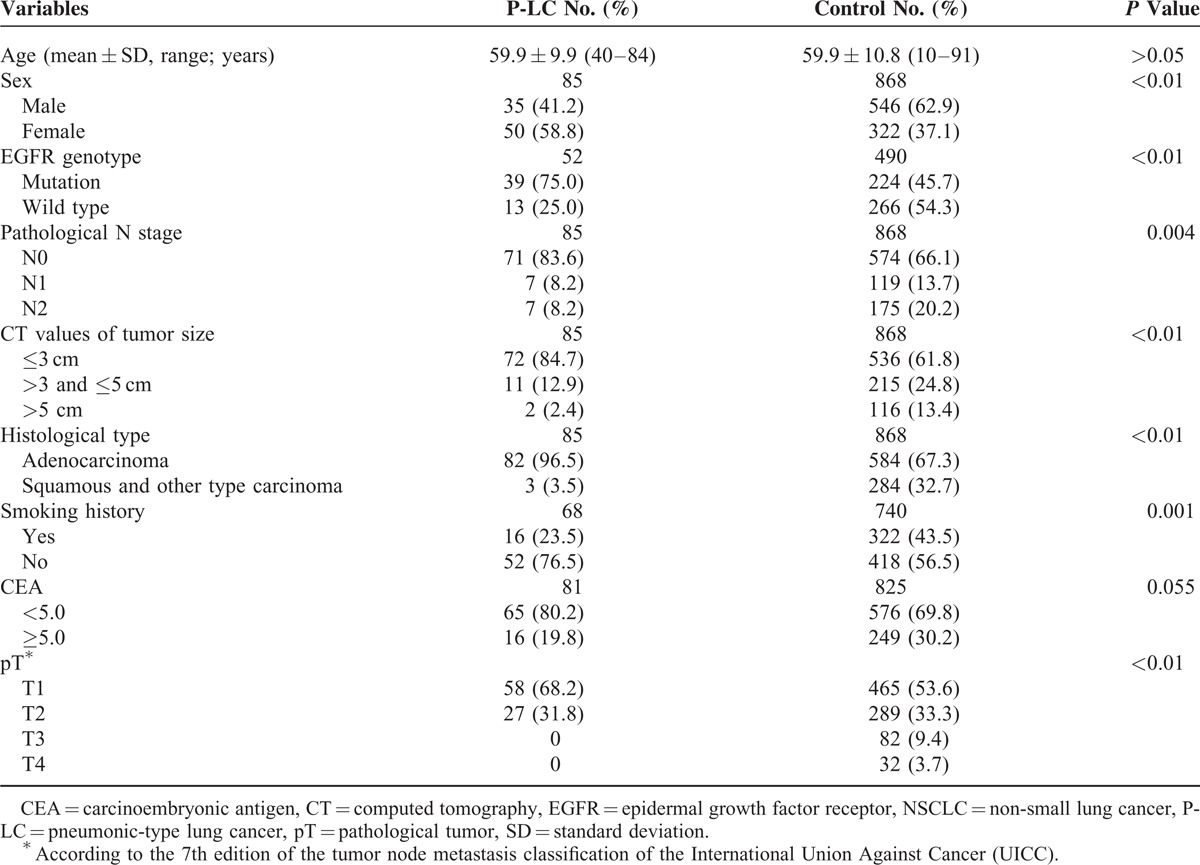
Clinical Features and EGFR Mutations in Patients with NSCLC and P-LC

The frequencies of tumor diameters of ≤3.0, >3.0, <5.0, and >5.0 cm was less in the P-LC group than in the control group (84.7%, 12.9%, and 2.4% vs 61.8%, 24.8%, and 13.4%, *P* < 0.01, respectively). The pathological tumor stage of T1,T2,T3, and T4 in the P-LC group was lower than the control group (68.2%, 31.8%, 0%, and 0% vs 53.6%, 33.3%, 9.4%, and 3.7%, *P* < 0.01). Pathological staging showed most cases were N0 in both P-LC (83.6%) and control groups (66.1%). However, significant difference was found on frequencies of lymph node metastasis between P-LC and control groups (83.6% vs 66.1%; *P* = 0.004).

### Pathological Results

The pathological classification of these 2 groups is shown in Table 2. There were 82 cases (96.5%) of adenocarcinoma in the P-LC group, this number was significantly higher than the control group: 584(67.3%) cases of adenocarcinoma, 2 cases (2.4%) of squamous cell, and 1 case (1.2%) other type. Further analysis showed no significant difference of adenocarcinoma subtype (invasive adenocarcinoma: 78/82 vs 520/584, *P* = 0.089; MIA: 4/82 vs 58/584, *P* = 0.104) between P-LC and control group. We performed the classification according to the new edition of pathological typing and found no significant difference in lepidic growth pattern, acinar growth pattern, solid growth pattern, papillary growth pattern, and variant invasive adenocarcinoma, between the 2 groups.

**TABLE 2:**
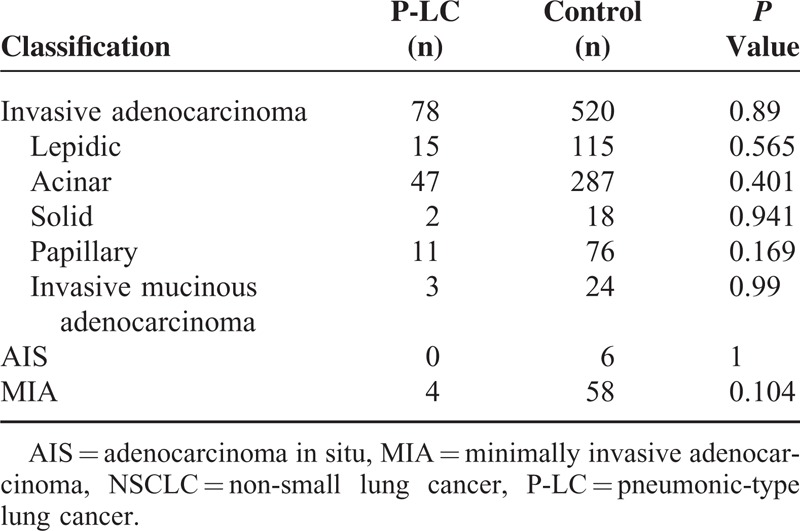
Classification of Lung Adenocarcinoma in Patients With NSCLC and P-LC

### EGFR Genotyping Results

EGFR mutation analysis was performed on 542 of the 953 patients; 52 of these were confirmed P-LC patients and EGFR mutation was confirmed in 263 (48.5%) patients. EGFR mutation was higher in P-LC patients than control group patients (75% vs 45.7%, *P* < 0.01). EGFR mutations between P-LC and control patients are shown in Table 3. A significant increase in the number of adenocarcinoma were found in P-LC group compared with the control group (75% vs 52.8%, *P* = 0.002). There was no significant difference on EGFR mutation type between P-LC and control group (exon 19 deletion: 21/40 vs 94/221, *P* = 0.243; exon 21 L858R/L861Q: 17/40 vs 118/221, *P* = 0.205; exon 20 insertion: 2/40 vs 4/221, *P* = 0.506; exon 18 G719X: 0/40 vs 5/221, *P* = 0.243).

**TABLE 3:**
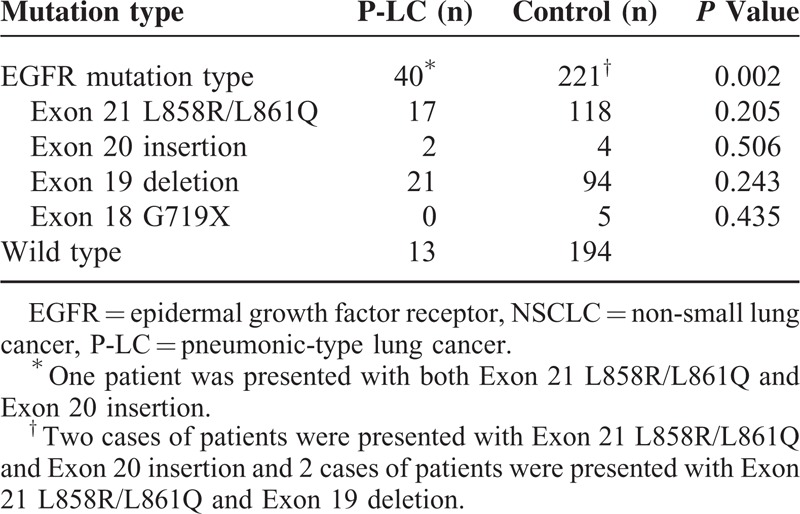
EGFR Mutations in Lung Adenocarcinoma of Patients With NSCLC and P-LC

### Characteristics of EGFR Mutations

All 542, dependents on EGFR mutations, patients were divided into 2 groups: EGFR mutation positive and wild-type group. The characteristics of these 2 groups are shown in Table 4. The results of logistic regression analysis showed histologic type (odds ratio [OR] = 12.681, 95% confidence interval [CI] = 4.936–32.578, *P* < 0.01), sex (OR = 2.168, 95% CI = 1.325–3.545, *P* = 0.002) and radiologic manifestation of P-LC (OR = 2.966, 95% CI = 1.318–6.672, *P* = 0.009) to be independent predictors of EGFR mutations, whereas smoking history and EGFR amplification method were not.

**TABLE 4:**
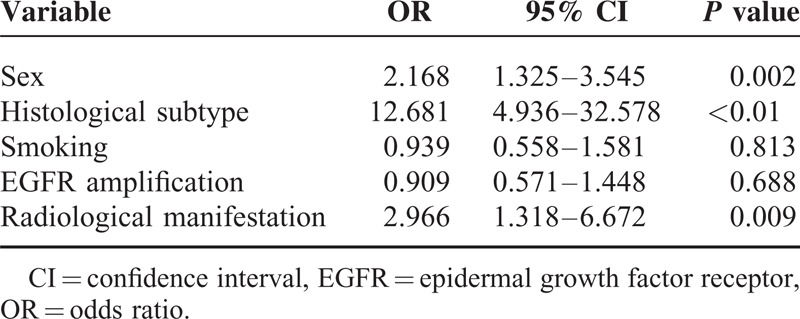
Relationship Among EGFR Mutation, Sex, Histological Subtype, Smoking, EGFR Amplification and Radiological Manifestations

## DISCUSSION

According to imaging manifestations, P-LC is a special type of inflammatory lung cancer, not classified by WHO and not commonly used in clinical practice. However, only 8.9% of patients with lung cancer confirmed through postoperation pathological analysis presented with inflammatory changes on CT findings, most patients presented with adenocarcinoma. Previous studies have shown that most P-LC cases to be adenocarcinoma, and the most common major pathological manifestation was bronchioloalveolar carcinoma (BAC) with a lepidic growth pattern.^4,15^ However, although P-LC has been identified in early reports, we found most cases of P-LC presented as invasive lung cancer according to the new diagnosis criteria of lung adenocarcinoma issued in 2011. Because of this, it is necessary to reexamine the histologic manifestation of P-LC.

According to WHO's diagnostic criteria, BAC is defined as neoplastic cells along preexisting alveolar structures (lepidic growth), lacking stromal, vascular or pleural invasion. It can be further histologically classified into mucinous and nonmucinous subtypes.^16^ However, the term BAC could be used in multiple types of adenocarcinoma, including small isolated peripheral adenocarcinoma, MIA, mixed-type invasive adenocarcinoma, and widespread advanced adenocarcinoma. Almost all mucinous BAC were invasive adenocarcinoma; these low or highly malignant adenocarcinoma could be misdiagnosed as “BAC”. The consequences of confusion from multiple uses of the term BAC in the clinical and research areas have been addressed, and therefore BAC was no longer used after the 2011 classification of lung adenocarcinoma and the term was further divided into AIS, MIA, and invasive adenocarcinomas classified by predominant pattern after using comprehensive histologic subtyping and invasive mucinous adenocarcinoma.^11^ We found only 4 cases of MIA in the P-LC group, the other 48 cases were confirmed invasive adenocarcinoma with 15 cases of lepidic growth pattern. In other words, only a small percentage of BAC (22.4%) patients were found in the P-LC group. This may be attributed to histologic subtyping. Acinar growth pattern was the predominant pattern in 60% of invasive adenocarcinoma patients in the P-LC group; 21.8% cases were lepidic growth pattern. More clinical research should be conducted to determine whether the lepidic growth pattern could be considered as the main type of P-LC.

As most of the P-LC patients were female and nonsmokers, similar to the clinical features of most EGFR mutation patients, we reviewed the EGFR mutation analysis in patients with P-LC. Among the 52 P-LC cases with postoperative EGFR mutation analysis, 39 (75%) cases presented with EGFR mutation, which was significantly higher than both the control group and frequencies of EGFR mutation (63.2%) in female and nonsmoker patients (cite). The logistic regression analysis found sex (*P* = 0.002) and P-LC (*P* = 0.006) to be independent predictors of EGFR mutation but not smoking history (*P* = 0.811). These results suggested that CT finding of P-LC was an indicator of EGFR mutation independent of sex, histologic type, and smoking history. EGFR mutation was closely correlated to the histologic subtype of lung adenocarcinoma. A recent study conducted by Yanagawa et al^17^ showed that the frequency of EGFR mutation was AIS (62%), MIA (60%), lepidic (77%), acinar (49%), papillary (50%), solid (28%), micropapillary (43%), and invasive mucinous adenocarcinoma (0%). The high frequency of EGFR mutation in P-LC may be caused by following: most cases were adenocarcinoma with lepidic and acinar growth pattern; the majority of patients were female; there was a high ratio of nonsmoker; and patients were Asian.

Early studies have shown that P-LC is a malignant cancer with poor prognosis, the 5-year survival rate was only 27%.^18,3^ Misdiagnosis is usually made because P-LC patients presented with normal radiograph of inflammatory changes.^15,19^ Wislez et al^3^ showed that 36.5% (19/52) of P-LC patients could be confirmed by postoperation histologic analysis and others could be verified by pathologic analysis through biopsy or bronchotrachoalveolar lavage fluid. Participants included in our study were NSCLC patients confirmed by histological analysis, and we found the rate of lymph node metastasis low in P-LC patients: 83.6% of patients were in N0 stage. For the patients with single peripheral P-LC, endobronchial ultrasound-guided biopsy and mediastinoscopy lymph node biopsy should be processed with caution.

Based on the above findings, EGFR mutation analysis was highly recommended for lung cancer patients with inflammatory features. For advanced patients, tyrosine kinase inhibitors could be considered an important option after clinical diagnosis when having difficulties obtaining pathologic specimens. High incidence of EGFR mutations were found in P-LC patients and systemic metastasis could coexist in the patients with EGFR mutation.^20^ Therefore, for potentially resectable disease, positron emission tomography/CT was suggested for the patients with similar lesions to identify the condition of systemic metastasis.

The limitations of this study include subjective criteria on the diagnosis of P-LC, selection bias due to the lack of advanced lung cancer patients, and lack of survival analysis due to the short period of follow-up.

P-LC patients showed a significant higher incidence of EGFR mutations, which was independent of sex, histological type, and smoking history. Patients with this type of CT finding EGFR mutation analysis should be performed routinely. However, the prognosis of P-LC patients requires further study.
